# Understanding contextual and practical factors to inform WHO recommendations on using chest imaging to monitor COVID-19 pulmonary sequelae: a qualitative study exploring stakeholders’ perspective

**DOI:** 10.1186/s12961-023-01088-1

**Published:** 2024-06-11

**Authors:** Gladys Honein-AbouHaidar, Cynthia Rizkallah, Imad Bou Akl, Gian Paolo Morgano, Tereza Vrbová, Emilie van Deventer, Maria del Rosario Perez, Elie A. Akl

**Affiliations:** 1 Hariri School of Nursing, American University of Beirut, Beirut, Lebanon; 2 Department of Internal Medicine, American University of Beirut, Beirut, Lebanon; 3 Department of Health Research Methods, Evidence and Impact McMaster University, 1280 Main Street West, Hamilton, Canada; 4 Czech National Centre for Evidence-Based Healthcare and Knowledge Translation (CEBHC-KT), Institute of Biostatistics and Analyses, Faculty of Medicine, Masaryk University, Brno, Czech Republic; 5 Radiation and Health Unit, Department of Environment, Climate Change and Health, World Health Organization, Geneva, Switzerland; 6 Department of Medicine, American University of Beirut, Riad-El-Solh, P.O. Box 11-0236, Beirut, 1107 2020 Lebanon

**Keywords:** COVID-19, Chest imaging, Practice guidelines, Qualitative research, Long COVID

## Abstract

**Background:**

A recommendation by the World Health Organization (WHO) was issued about the use of chest imaging to monitor pulmonary sequelae following recovery from COVID-19. This qualitative study aimed to explore the perspective of key stakeholders to understand their valuation of the outcome of the proposition, preferences for the modalities of chest imaging, acceptability, feasibility, impact on equity and practical considerations influencing the implementation of using chest imaging.

**Methods:**

A qualitative descriptive design using in-depth interviews approach. Key stakeholders included adult patients who recovered from the acute illness of COVID-19, and providers caring for those patients. The Evidence to Decision (EtD) conceptual framework was used to guide data collection of contextual and practical factors related to monitoring using imaging. Data analysis was based on the framework thematic analysis approach.

**Results:**

33 respondents, including providers and patients, were recruited from 15 different countries. Participants highly valued the ability to monitor progression and resolution of long-term sequelae but recommended the avoidance of overuse of imaging. Their preferences for the imaging modalities were recorded along with pros and cons. Equity concerns were reported across countries (e.g., access to resources) and within countries (e.g., disadvantaged groups lacked access to insurance). Both providers and patients accepted the use of imaging, some patients were concerned about affordability of the test. Facilitators included post- recovery units and protocols. Barriers to feasibility included low number of specialists in some countries, access to imaging tests among elderly living in nursing homes, experience of poor coordination of care, emotional exhaustion, and transportation challenges driving to a monitoring site.

**Conclusion:**

We were able to demonstrate that there is a high value and acceptability using imaging but there were factors influencing feasibility, equity and some practical considerations associated with implementation. We had a few suggestions to be considered by the expert panel in the formulation of the guideline to facilitate its implementation such as using validated risk score predictive tools for lung complications to recommend the appropriate imaging modality and complementary pulmonary function test.

**Supplementary Information:**

The online version contains supplementary material available at 10.1186/s12961-023-01088-1.

## Background

The novel coronavirus disease (COVID-19) first emerged in December 2019 in the city of Wuhan in China and became a pandemic as of March 2020. As of December 4, 2022 it had infected more than 611 million individuals and claimed the lives of almost 6.6 million individuals globally [40]. Ever since the first cases started to appear, the scientific community generated evidence related to the epidemiology of the virus, the clinical management of the disease, and its sequelae among survivors.

A systematic review of data from November 2021 till April 2022 showed that 32% of individuals affected by COVID-19 were asymptomatic [31]. A report from the Chinese Center for Disease Control and Prevention in 2020 showed that among those who developed symptoms, 40% had mild symptoms (fever, cough, fatigue, and headache) [41], mostly treated at home unless they developed complications that required short-term hospitalization. The remaining cases were treated in hospitals and encompassed moderate symptoms leading to pneumonia reported by 40%; severe symptoms that required oxygen therapy reported by 15% of the cases [39]. Finally, 5% develop complications leading to organ failure, mainly respiratory failure. Most of these complications increase in the presence of pre-existing risk factors [36].

Since December 2019 till November 2022, COVID-19 survivors account for more than 635 million individuals thus far. Many survivors experience sequelae of the illness that can linger for months, and are often referred to as “long-haulers” or sufferers of long-COVID syndrome [22, 25, 36]. Among those who recover from moderate or severe illness, long term pulmonary sequelae may be expected. The damage incurred by the virus during the acute and post-acute phase, causes the lung tissue to become fibrotic [4]. Other long-term consequences have been reported, including psychological [37], neurological, cardiovascular, and musculoskeletal, among others. Nalbandian et al. use the term “post-acute COVID-1” for long-term complications and/or symptoms present beyond four weeks from the beginning of the symptoms, i.e. after the acute phase of the disease [24]. The term “Post COVID-19 condition” has been initially proposed by WHO [6] and its clinical case definition was published [33].

Given the magnitude of this pandemic, the epidemiological studies that show the sequelae of the infection are important [22, 25, 36], but equally important is to know how to monitor those patients for those sequelae. The World Health Organization (WHO) has regularly updated guidance for clinical management of COVID-19, proposing a multidisciplinary approach to patient care after acute illness [39]. Early on in this pandemic, WHO issued a rapid advice guide on the use of chest imaging in acute care for COVID-19 patients considering three imaging modalities: radiography, computed tomography and ultrasound [1]. WHO conducted new systematic reviews [42] to update this rapid guidance and included a new research question on the use of chest imaging in patients who recovered from COVID-19. Persistence of pulmonary sequelae among COVID-19 patients are important to consider. Long-term physical, functional and neuropsychological complications resulting from the lung injury have been reported six to 12 months after discharge from hospital using chest imaging [8]. All severe cases admitted to intensive care units and 21% of moderate cases admitted to regular units had persistent pulmonary sequelae, which were detected using chest radiography imaging 8 weeks after discharge. 7% of patients showed glass opacities, defined as an area of haziness through which vessels and bronchial structures may still be seen, using computed tomography 18 weeks post-discharge [28]. One year after discharge, the prevalence of ground glass opacities dropped significantly but 95% of COVID-19 patients admitted to ICU had abnormal CT scans [8]. Hence, the importance of monitoring the sequelae of the infection. However, monitoring and compliance can be influenced by several other factors including availability of resources, transportation, co-morbidities, and other practical factors [15].

To optimally inform the development of this recommendation, it was considered important to explore qualitatively the relevant contextual factors influencing its implementation. WHO recommends using evidence from qualitative research to understand the valuation of the recommendations, their acceptability to stakeholders, their feasibility and equity, and the practical considerations influencing their implementation [38].

Thus, the aim of this study was to explore the perspective of key stakeholders, including providers and patients from the global community, to understand their valuation of the outcome of the proposition (i.e., monitoring pulmonary sequelae), their preferences for the modalities of chest imaging, and the acceptability, feasibility, impact on equity, and practical considerations influencing the implementation of using chest imaging.

## Methods and materials

### Design and approach

This study draws on a descriptive qualitative design [30] using an in-depth interview with key stakeholder informants.

We secured ethical approval from the American University of Beirut Institutional Ethics Board before starting the study. All participants provided a verbal informed consent to participate and record the discussion. We ensured confidentiality by anonymizing transcripts, assigning each participant a codename that starts with the country name followed by P for health care professionals and Pa for patients.

### Conceptual frameworks

We structured the study based on the GRADE Evidence to Decision (EtD) framework (see Table 1) [3], and on Heen et al. practical issues framework (see Table 2) [14]. We judged some practical issues to be already captured by the EtD framework (e.g., medical routine, procedure and device, recovery and adaptation, adverse effects, overall physical well-being, and costs and access). We judged other practical issues to be irrelevant (e.g., food and drinks, exercise and activities, social life and relationships, and work and education). Consequently, we deemed the following five practical issues as relevant: tests and visits, coordination of care, emotional well-being, pregnancy and nursing, and travel and driving.

**Table 1:** Summary of the EtD framework constructs and a short description of each

Evidence to Decision framework and practical issues	
Construct	Short description
Values	*Stakeholders’ attitudes towards and value placed on the intervention as well as perception of the importance of the intervention*
Preferences	*Stakeholders’ perception of the advantage of implementing one intervention versus an alternative*
Equity	*Stakeholders’ concerns that there may be groups or settings that the intervention may put at a disadvantage*
Acceptability	*The extent to which that intervention is considered to be reasonable among those receiving, delivering or affected by the intervention*
Feasibility	*The likelihood that the intervention can be properly carried out or implemented in a given context*

Alonso-Coello, P., Schünemann, H. J., Moberg, J., Brignardello-Petersen, R., Akl, E. A., Davoli, M., et al. (2016). GRADE Evidence to Decision (EtD) frameworks: a systematic and transparent approach to making well informed healthcare choices. 1: Introduction. BMJ, 353. doi: https://doi.org/10.1136/bmj.i2016

**Table 2:** Twelve key categories of practical issues important to inform decision-making

Medical routine	Adverse effect, interactions and antidote	Food and drinks
Tests and visits	Physical well-being	Exercise and activities
Procedure and device	Emotional well-being	Social life and relationships
Recovery and adaptation	Pregnancy and nursing	Work and education
Coordination of care	Costs and access	Travel and driving

Heen, A. F., Vandvik, P. O., Brandt, L., Montori, V. M., Lytvyn, L., Guyatt, G., et al. (2021). A framework for practical issues was developed to inform shared decision-making tools and clinical guidelines. Journal of Clinical Epidemiology, 129, 104–113. https://www.jclinepi.com/article/S0895-4356(20)31141-0/pdf

### Sample and recruitment

We used the purposeful sampling approach. Our key informant group included adult patients (18 years of age or older) who have recovered from acute COVID-19 illness. We also targeted health practitioners, specifically radiologists and pulmonologists caring for those patients. We excluded providers not involved in the care of COVID-19 patients.

We aimed to adopt a maximum variation sampling approach in terms of severity of disease for patients (mild, moderate, or severe) and job title for practitioners across all regions of the world.

At first, we identified health care professionals in each of the six WHO regions (Africa, Americas, South-East Asia, Europe, Eastern Mediterranean, and Western Pacific) through health care providers on the research team (known sponsor sampling approach). [MQ, 1988. #27] Then, we asked the identified providers to invite patients from their practice to participate in the study (snowball sampling). We also asked patient advocates from different countries to identify potential participants, who were invited by email until saturation was reached. (MQ., 1988).

### Data collection

We used a semi-structured approach for interviews using both predetermined open-ended and probing questions (Additional file 1: Appendix 1: KI interview guide for providers and Additional file 2: Appendix 2: KI interview guide for patients) [7]

The interviewer (GHA), an experienced qualitative researcher who had no prior relationship with the participants, conducted the virtual interviews using WebEx. Interviews lasted on average 60 min (range 30–90 min). The discussions were in English, except for one interview conducted in Italian and translated by GPM.

We recruited between October 27, 2020, and December 26, 2020, a total of 33 participants, including 10 patients and 23 providers (of which 11 pulmonologists, 5 radiologists, 3 nephrologists, 2 intensivists, 1 family physician and 1 general practitioner) (Additional file 3: Appendix 3: Participants’ list: providers and patients).

The first author conducted all interviews virtually using WebEx, Zoom or WhatsApp applications. Participants chose the location at the time of their preference. Recruitment and data collection occurred between October 27 and December 26, 2020.

### Data analysis

GHA and CR conducted the analysis using the framework thematic analytical approach. This analytical approach consisted of 7 stages [12]. In stage 1, CR transcribed the audio-recordings verbatim. In Stage 2, GHA and CR familiarized themselves with the content of the discussion by reading each transcript and taking notes. In stage 3, CR indexed the data based on the GRADE EtD construct and Heen et al. practical issues. She provided a label for each meaningful datum. In stage 4, after indexing a few transcripts, GHA and CR met to discuss their labels and merge them into categories. In stage 5, CR applied those categories to the remaining transcripts. In stage 6, GHA and CR charted all opinions and views indexed under each category. In stage 7, they compared and contrasted the categories, and mapped connections between them. Finally, a complete narrative of the findings was created. These findings were supported by quotations triangulated between health care professionals and patients.

We ensured credibility, reflexivity, and confirmability throughout this process. For credibility and confirmability we used transcribed audio-recorded interviews as the main data repository maximum variation sampling, and a semi-structured interview approach. For reflexivity, the interviewer had no prior relationship with participants and two individuals were involved in the analysis to avoid bias interpretation of the results.

The reporting of this study followed the Consolidated criteria for Reporting Qualitative research (COREQ) Checklist [35] (Additional file 4: Appendix 4: COREQ checklist).

## Results

Table 3 provides a summary of results for the perceptions of stakeholders of the contextual factors, based on the GRADE EtD framework and Heen et al. practical issues.

**Table 3:** Summary of findings for the perceptions of stakeholders of the contextual factors based on the EtD framework related to the use of chest imaging to monitor pulmonary sequelae following recovery from COVID-19 illness

Evidence to Decision framework and practical issues		
Construct		Findings
EtD		
A	Values	*Monitoring COVID-19 patients post-recovery from an acute episode using radiation was highly valued by patients and providers when chosen wisely* *Non-ethical where there is no clinical indication*
B	Preferences	*Using the right test for the right indication was a very important criterion for decision-making. However, there were pros and cons for each imaging modality*
C	Acceptability	*Among providers, there was convergence that monitoring using imaging is a common practice well received by patients* *Among patients, the fact that some were already accustomed to the monitoring approach for chronic diseases, for example, HIV and tuberculosis co-infection, monitoring was acceptable* *Affordability was a deterrent*
D	Equity	*Source of inequities can be grouped into two categories:* *Across-country (global) inequity: access to resources varied by country (global south versus global north); and universal versus partial health insurance coverage as determinant for financial access to services)* *Within-country inequity: some disadvantaged groups lacked access to private insurance; the quality of public services for the non-insured was lower than that of private services for the insured; those living in urban areas were at more of an advantage than those living in rural areas; and non-COVID-19 patients were at a disadvantage due to treatment deferrals*
E	Feasibility	*Feasibility of monitoring was facilitated by the following:* *Having access to a post-recovery COVID-19 units and protocols* *Annexing chest imaging monitoring to an already existing monitoring system for other diseases* *Well-coordinated team willing to scale up the efforts to meet the demand* *But the barriers are:* *Severe cases being extremely deconditioned, thus several visits cannot be paid* *Low number of specialists in some countries* *Limited number of staff responsible for tracing patients* *Limited number of equipment (for example, CT scanners)*
Practical issues		
1	Number of visits	*Challenging for elderly individuals living in nursing home and those dependent on others* *Dialysis patients*
2	Coordination of care	*Lack of coordination as shown in the multiple phone calls related to the same issue*
3	Emotional well-being	*Psychological stress while waiting for further results*
4	Travel and driving	*Out-of-pocket cost* *Burden on dependents*
5	Pregnant and nursing mothers	*Extra precautions are need to avoid harm*

HIV, human immunodeficiency virus

In the following text, we present a detailed narrative of themes illustrated by selected quotes from participants. In Additional file 5: Appendices 5–10, we provide exemplary quotes from both providers and patients for each theme.

A. Monitoring progression is highly valued when chosen wisely and acceptable.

All patients thought if their providers asked them to do chest imaging post-recovery, they would not question its value and they “*would do whatever they [providers] wanted” ****US-Pa01.***

Similarly providers perceived imaging post-COVID-19 as highly valuable when there is clinical indication. Mild cases ‘*patients don’t routinely need any kind of follow up imaging*’.***US-P03.***

While moderate and severe cases as well as those with underlying chronic diseases such as human immunodeficiency virus (HIV) infection and tuberculosis coinfection, it will be highly valued, as this participant indicated:

***India-P13:**** So it depends on the severity of the disease during the hospitalization, and the condition of the patient on discharge.*

Further, COVID-19 patients, particularly those who were hospitalized might end up with potential multi-organ dysfunction, and the stay in the intensive care unit might expose them to various diseases, such as pneumonia. Hence, for many providers monitoring them post-recovery would be an opportunity to screen and intervene on time to avoid further complications. For example, this provider pointed to low-immunity and co-infection and the importance of frequent monitoring on the treatment.

***Nigeria- P06:**** The follow up is major. Some of them have a low immunity and if we do not catch the bacterial infection on time yes, we lost them. I think the follow up is very crucial.*

Interestingly, one provider indicated that, given that this is a new disease, monitoring patients post-recovery can help the scientific understanding of the long-term sequelae of the infection and eventually improve the care.

***US-P08****: mostly to understand the disease more. You see what they have findings on the imaging initially they have been improving.**** Also mostly to kind of look at long term sequela of the condition. But mostly to understand the disease itself, which is kind of relatively had been new.***

On the other hand, a few provider participants cautioned against over-testing. First, it would be unnecessary since it will not add *“to the decision making in terms of therapy…[and] ****you**** don’t want them to be having unnecessary exposure to radiation with multiples radiographs or multiple CT scans. ****India-P09.***

Further, over-testing might end up detecting indolent findings, which would add to their psychological stress, hence unethical from the perspective of one participant:

***Swiss-P02:**** “I think so, and then you might have a problem that you start to find incidental findings. And then you’re going to have to deal with following up on other things that you might see on the X ray that may not be even real, you know, and that’s another ethical problem. If they don’t need the radiography, you’re exposing them to radiation*This psychological stress to wait yet for other results depleted patients’ tolerance. Who indicated that they “ *would be very anxious because... my lung function might be deteriorating, I might have long term sequela.**** Ethiopia-Pa03.***

B. Monitoring and follow-up are a reasonable clinical practice.

Among providers, we found that monitoring using imaging to follow-up on the patient’s resolution, or any potential sequelae of the disease was an acceptable practice, in fact it has been already a common practice among almost all participants except for one participant who indicated that due to poor resources in the country, monitoring was never an option.

***Cameroon-P11****: No monitoring whatsoever ... Okay so I would think it’s very important to do radiological follow up on them specially to see how much damage has occurred in their lungs and also to see if some damages are still continuing after discharge. In essence our potentials in treating COVID- 19 does not lay so much emphasis on radiological findings and I understand that this is a limitation of our treatment.*

Providers also indicated that since some COVID-19 patients were already accustomed to monitoring for other underlying diseases, such as for dialysis or cardiac matters, adding chest imaging monitoring would be acceptable for them. However, the downside is that often times patients may need to spend longer time for the visit, which can be beyond their ability to tolerate.

***Swiss-P02:**** Well, I have to say if a patient is on dialysis, and they have to come 3 times a week. So, we see them and …we try to do a radiography at the baseline… we do another radiography or a CT or whatever but basically for us we get to see them pretty routinely... I think for the dialysis patients again, for them, it’s a burden because either they must come earlier to go to the radiography before the dialysis, or then go after dialysis and then it delays them going home. And then it complicates the transport.*

Patients found monitoring a reasonable process, however the cost of the test would matter.

***Africa-Pa02:**** No because it [test] is very expensive, both CT scan and treatment*

C. Preferences for each chest imaging modality are driven by indication and the pros and cons of each.

Preferences for the different imaging modalities were mainly voiced by providers. The clinical indication and the pros and cons for each test dictate the type of test to be done.

Chest radiography is the preferred option for the following conditions: mild cases still complaining of chest pain to monitor clearance of the lungs, and for dialysis patients to detect water retention as one nephrologist indicated:

***Swiss-P02:**** I think honestly, for our patients, sometimes the X rays actually helpful. Because sometimes they lose a lot of weight, and we need to know how much water we need to remove from the patient in a dialysis treatment. And occasionally you don’t know how quickly the patient has lost weight with COVID. So, sometimes you do the X Ray with an excuse of COVID, but you’re actually looking to see, are they filling up with water, are they’re going to develop heart failure. So, for us, sometimes there’s another extra added value.*

There were a few pros for using X-rays including ‘*reducing the radiation from CT [which] is so large that if you do so frequently, it may not be good or ethical’* US-P07. It is available in local clinics as one provider indicated: *‘a lot of family doctors can do X rays. It’s amazing. They have an X ray thing in their clinic in their little office’* USP02. At times the choice of the test is driven by the cost of the test, therefore providers would choose whatever is affordable for patients as this provider indicated: ‘*The common is Chest X ray… but in some cases we do high resolution CT because CT is expensive so we do it for only those who can afford it, not for all of them’* **US-P06.**

The cons are not being sensitive enough and may miss significant pathology.

*‘The chest X Ray, it’s not sensitive enough to show you a significant pathology. And so we have seen it with COVID, you know the chest X Ray misses a proportion, like, 40% or something’ ****US-P07.***

Providers gave several indications for choosing chest CT scan. Chest CT scan is very sensitive to ground glass, consolidation, and fluid retention. It is also recommended for patients still depending on oxygen long after discharge. Often, it is used in cases where providers are anticipating persistent interstitial manifestations like non-specific interstitial pneumonia (NSIP). It can identify fibrotic changes and detect additional diseases, if any.

***India- P05:**** as far as the CT scan is concerned, it is basically, it gives you a fuller picture of the chest. I mean, what is happening inside the lungs, right from the trachea to the lung parenchyma. So generally, the mindset for patients out here is better go for a CT scan because, uh, it gives a good resolution.*

The cons included higher radiation exposure, lack of accessibility in local clinics and affordability for various populations.

*‘If you want a CT, then the patient has to go to a hospital or a private place and then definitely for some of them it involves travel and sometimes it’s even hours of travel’ ****US-P02****.*

The nursing and pregnant women population was of concern for radiologists, as extra precautions are needed to avoid any harm when doing the imaging.

***South Africa- P16:**** So, high radiation risk or high radiation dose to the breasts. And reasonably achievable. So, we try to take it down to the minimum.*

Finally, for the lung ultrasound, it is the preferred option to assess peripheral consolidation. It is considered an easy modality test as it is portable, accessible in local clinics, and affordable.

***US-P08:**** … We’ve been relying a lot on it and they’re criteria to follow with the ultrasound. It’s a very easy modality. Yes, we’re using it. And now it’s like, you have those small portable ones. It’s very easy to clean it from room after room, way better than cleaning the whole chest X Ray machine.*

The potential for missing central consolidation and significant pathology were cited as the major cons.

*‘I mean, scanned by ultrasound, we are only able to see the peripheral one third, which are involved in COVID, but it is not only the peripheral. So central areas, we are not able to visualize using ultrasound’ ****US-P05.***

D. Equity concerns across countries and within countries.

The availability of resources and health insurance coverage across and within countries impacted equity. Access to resources varied between developed such as USA and developing countries such as African countries.

One provider from the USA said:

***US-P08:**** We have 2 big centers. CT scan is available very widely. Any issues that need any further care, again, we have video system, so if they need further evaluation, they go to their closest hospital, they get stabilized and they get transferred to one of the biggest centers, which is not too far. And either they get transferred by ground, or they’re sick, they get transferred by air, which is really fast, like talking 10 to 15 minutes by helicopter or a plane. So, the CT scan is widely available if it needs, like, a little bit of more advanced things the biggest centers, definitely, the patients get referred to them, but mostly when they get sick and need admission. For outpatient, like, imaging, scans, pulmonary function test, ultrasounds, these things are very widely available.*

Compared with what providers in Africa said:

***Cameroon-P11:**** Okay so I would think it’s very important to do radiological follow up…Most of our district health facilities will not have radiology equipment. So normally we would just do auscultations, and manage them as such, and when they go, we do not follow up.*

***Ethiopia-P07:**** We have only one CT scan room, so we don’t want to contaminate that. We have a lot of patients, so we only order chest radiography using a machine which is dedicated for that purpose. So definitely if you ask the people to take CT scan for all those who have COVID, it will definitely it would put a burden to the imaging service we have and also to the health professionals, and they think that they can also have COVID-19.*

One patient said when the cost of the test is fully covered, everyone would comply.

***Czech-Pa06:**** it’s covered by the health insurance. Okay. So everyone is covered, everybody in the Czech Republic*

But when there is no insurance coverage, then patients would more likely opt out.

***Pakistan-Pa04:**** I am a teacher, so my insurance company is taking care of my entire cost of stay in hospital and all expenses…Not the case for everyone... We are a third world country. For example, Remdesivir many cannot afford it. CT scan is slightly expensive. 75% will not be able to afford those expenses.*

Other factors impacting equity include the quality chasm between public and private within a country. Often, the non-insured have to rely on public services, which are typically described as crowded, under-resourced, and generally of poorer quality compared to private ones. Similarly, patients living in urban areas have better accessibility to services as compared to patients living in rural areas.

***India- P09:**** So, CT scan facilities are most of the time available in, at least 2 or 3 cities like ours. Public sector it is a few. It is difficult to access. In private sectors, there are lots of CT scans, but again, the out of expenditure really goes high.*

***India- P05:**** Yeah, that can be a problem because the means of travel are, you know, buses and trains in our country for traveling from one area to another and areas who have radiological imaging modalities are mostly located in urban areas and suburban areas.*

Finally, non-COVID patients were perceived to be at disadvantage during the pandemic. Despite their needs for follow-up, many were either avoiding health care institutions (out of fear of getting the disease) or were being deferred by their providers to avoid exposing them to the infection. In either case, their follow-up care was being jeopardized by the pandemic.

***India-P09:**** Yeah, so definitely COVID patients are still able to access healthcare in an appropriate period. And it is most unfortunate, those patients who have non-COVID illness, they are much more neglected, uh, because of this whole crisis.*

E. Barriers and facilitators for feasibility.

Providers reported three facilitators of feasibility. Having a post-recovery COVID-19 unit with protocols was the main lever for providers to act. A provider from Ethiopia said:

*‘We have a clinic, which is, uh, you know, we have a head nurse. We have a few. 6 to 7 nurses and because we have limited number of nurses, this group of nurses, half of the time they spend it with the chest team… When they come to the clinic, and we see like, 45 to 50 patients and that divided to like on average 5 residents, 1 fellow and 1 senior. We have to go through each CT scan of each patient’ ****Ethiopia-P07.***

Second, many health centers had already established monitoring systems for other diseases, e.g., kidney disease requiring dialysis. Having the infrastructure already in place made it easier for them to build on it by adding the monitoring process for COVID-19.

*‘It will actually be going to be easily integrated because a lot of these patients [oncology patients] they end up getting CT scans and, you know, pet scans routinely. Yeah, because as part of their, you know, follow up and as part of their staging, so I don’t think it will be a problem at all… What we can do, and we’ve been doing that is to try and combine these tests together so that if they have 1 appointment, they can get the rest of it together’ ****Jordan- P04.***

The third facilitating factor was the ability to scale up team efforts. Dedicated teams were able to provide optimal services despite the multiple challenges as illustrated in this quote:

***India-P05:**** Daily, we are able to see around 75 to 100 patients…what we have done in our Institute, that we have a hand, picked a few consultants from my department myself included. So, every other day, and, I mean, every alternate day, we are having emergency duties. So we are having dedicated duties for reporting COVID patients, on CT scan and we do it, uh, every alternate day…I mean, it has increased the burden for sure. But then we are doing it.*

But the reported challenges were numerous too.

Most providers from low- and middle-income countries indicated that their human and non-human resources were limited. They did not have enough specialists in the country to do the monitoring, including pulmonologists and radiologists.

*‘The lack of specialists to follow these patients. I mean, South Africa is a country of 65M people, and we’ve got 70 pulmonologists’**** South Africa-P14.***

For both developing and developed countries, the number of support staff available to do the tracing did not commensurate with the need, given the large number of patients.

*I think the biggest challenge, obviously there’s a huge number of patients. It is overwhelming like, we’ve created a small group of doctors to, like, call patients and tell them they’re COVID positive and discuss the results. But eventually we were overwhelmed because there just so many positive patients. And so the solution was to then just say, well, you should talk to your ordering provider, your primary provider. The hard part there is as much as everyone is trying to keep up with, things changed so quickly with COVID recommendations, that primary care providers who are giving the right advice 6 weeks ago are no longer giving you the right advice ****US-P03.***

The back log in imaging appointments due to limited number of equipment (e.g., CT scan) challenged the feasibility in developing countries.

***‘****Yeah, we have a back log. We have a lot of back logs [radiology tests]. Even for admitted patients, it is difficult sometimes’ ****Ethiopia-P11***

Some providers indicated that for elderly population, especially those living in nursing homes, access to imaging would be a challenge due to imposed COVID-19 restrictions.

***US-P03:**** The patient population, that’s a little harder to get imaging on actually patients who go to, like, nursing homes. Because many skilled nursing facilities, they don’t have radiology services there and the ability to get the patient with medical transport from the skilled nursing facility to wherever the radiology is in back or the doctor’s appointment, or whatever, that too is also actually very difficult. So I think that population, that group where they have both mobility and transportation issues is a big one*.

Patients reported different feasibility challenges.

One patient who was a severe case indicated that he was extremely deconditioned after discharge. If it was not for the rehabilitation, he would not be able to do any further testing.

***US-Pa01****: I had to be lying in bed for 3 weeks. So after that, I literally could not stand up. I wasn’t able to sort of get out of the bed into a wheelchair to go to the bathroom for a week. We’re talking about a severe case.*

Coordination between health care providers was lacking. For example, one patient reported receiving phone calls from the primary care provider, from their pulmonologist and another from the health center, related to the same issue.

***Ethiopia-Pa03:**** Yeah, I think so. From my side, I was communicating with the social worker from the hospital because I was a staff there, so they call me, and they put me in contact with the psych department…. Things are very decentralized so a lot of guys might call them. The follow up is not organized so this might cause people to avoid seeking health care.*

For some, it was too much to bear, too many calls, too many follow-ups.

***Swiss-P02****: I think they get frustrated because they’re being phoned by a lot of different people. There’s quite a lot of phone calls also by people from the community checking on them and the officials checking that they’re at home and not outside when they should be at home, you know, these things. So I think they get a lot of phone calls. And I know for some of them, it’s definitely quite burdensome yeah, yeah.*

## Discussion

This study highlighted important factors that informed the development of the WHO recommendation about the use of chest imaging for monitoring patients who recovered from COVID-19. While WHO suggests not systematically scheduling chest imaging follow-up at the time of hospital discharge, it identifies patient groups who might benefit from periodic follow-up imaging. This study showed that providers from 15 countries highly valued this recommendation and that patients did not question its value if it was requested by their providers. Some feasibility challenges related to availability of human and non-human resources (e.g. lack of specialists, supporting staff and/or radiological equipment), represented major challenges for implementation and likely led to inequities between high and low resource countries. Lack of specialists, support staff and radiological equipment were the major challenges for implementation.

Using chest imaging to assess lung sequelae evolution and early detection of complications such is unquestioned [17]. We know by now that COVID-19 survivors’ number is in the hundreds of millions worldwide, and the figures continue to rise. We also know that residual lung complications including lung fibrosis, bronchiectasis, or other structural abnormalities [18, 19] are expected to remain months post-discharge among three quarters of patients [43]. Those sequelae affect lung functioning, exercise capacity and health related quality of life [18, 19].

However, we anticipate that the monitoring using imaging on this gigantic number of survivors is far from being a simple one size fits all approach. As noted in this study, variation in resources mattered. Thus, we propose a few suggestions to facilitate the implementation of the recommended guideline, and we discuss them below. First, clinicians can include risk stratification based on validated risk score predictive tools for lung complications to recognize the patients who might benefit from follow-up imaging and identify the appropriate imaging modality, hence reducing unnecessary tests and avoiding unjustified radiation exposure, at times unethical as suggested by our participants. In low-resource settings where CT scans are scarce, effective evidence-based alternatives for imaging can be used. Third, complementary lung function tests can be concurrently considered to reduce unnecessary use of imaging. Fourth, rehabilitation programs for COVID-19 survivors are needed for monitoring patients.

Triaging patients into average and high risk for lung complications can be done using several tools from as simple as a risk score predictive tool, [17, 21] imaging scoring to a more sophisticated artificial intelligence tool [10, 11]. In either case, those tools are often based on key indicators that are meant to predict lung complications and to aid clinicians in their decision-making [13, 21]. In this study, most providers were already stratifying patients based on the severity of the case during the acute phase. It is possible that their decision-making was arbitrary thus running into the risk of introducing algorithmic bias. To alleviate those systemic errors, the validated predictive risk score, imaging scores, or AI tools are meant to alleviate these bias and systemic errors. Examples of tools are numerous. Liang et al. as an example of predictive risk score, encompassed age (more than 65), co-morbidities, and Lactate dehydrogenase, blood urea nitrogen, D-dimer, procalcitonin, and ferritin levels provided an efficient risk evaluation system, and the model had an excellent discrimination and calibration during internal validation [21]. Chest radiography scoring systems and High Resolution CT scan categorization and quantification have also been used to stratify patients’ severity. [17] Alexander et al. reported a number of AI algorithms (more than 75) related to pulmonary medicine that received US Food and Drug Administration and Conformité Européenne approval [2]. It is warranted to increase awareness and adoption of those tools among providers in this specialty [2].

The role of the different modalities of imaging in the acute phase is more commonly reported in the literature than in the post-recovery phase [5, 16, 17]. Given the insufficient historic data on the sequelae of COVID-19, it is understandable that the evidence is still scattered and scant around the capabilities and limitations of each imaging modality in monitoring sequelae post-recovery. Shaw et al. indicated that chest radiography can be used for follow-up imaging despite the limitations in detecting subtle parenchymal changes, while chest CT is a more sensitive modality [32]. Lung ultrasound is also another option to evaluate sequelae [5]. Peng et al. found that US findings correlate with typical CT findings specifically useful to identify peripheral distribution of lung involvement [27]. Hence, the choice of modality needs to be carefully considered based on the clinical presentations and the availability of resources. However, more longitudinal studies are needed to refine this evidence and guide the practice (Rubin A, 2012) and systematic reviews of the literature are also needed.

The sequelae of COVID-19 need also to be assessed using complementary testing. Despite being asymptomatic, residual abnormalities in pulmonary function tests are expected weeks and months after discharge. Patients with persistent, new, or changing symptoms should have access to follow-up care including screening, assessment and rehabilitation interventions comprising pulmonary function tests when resources permit [39]. Zhao et al. found that the diffusion capacity of the lung for carbon monoxide (DLCO) measured by means of breath spirometry test can detect abnormal lung function among survivors. Urea nitrogen is another parameter to detect residual changes after discharge. D-Dimer elevation was an independent predictor for abnormal DLCO, thus patients with raised D-Dimer require pulmonary rehabilitation and need to be followed-up for sequelae [43].

Potential diagnostic testing strategies to inform clinical care and prognosis in the Post COVID-19 condition are numerous. New insights on the multidisciplinary nature of this condition suggest that several imaging modalities—in addition to chest imaging—might be considered subject to the clinical condition [26].

A few of our providers reported having rehabilitation centers for COVID-19 patients. Two of the severe cases we interviewed reported being severely deconditioning after spending on average three weeks in the intensive care unit, one reported being transferred to a rehabilitation center. Thornton indicates that despite the severity of their illnesses, COVID-19 patients unlike heart attack, trauma, and stroke, leave hospitals with the least support. Further, an estimated 45% of patients may require low level of rehabilitation interventions, while 4% will require more intensive rehabilitation for their neurological, respiratory and mental sequelae [34]. When asked about the feasibility of doing the imaging, some said they were too tired to go out of their homes. Hence, the need for rehabilitation programs in each country is a necessity.

The findings of this qualitative study were presented to the expert panel developing the WHO recommendation on the use of chest imaging to monitor COVID-19 pulmonary sequelae. The expert panel did consider the important contextual and practical factors influencing the implementation of the recommendation when issuing their recommendation.

### Strengths and limitations

There are areas of strengths and a few limitations to be acknowledged. The most important strength is the qualitative approach adopted to delve into the details influencing the implementation of the guideline from the perspective of the stakeholders. Primary qualitative studies or systematic reviews, called qualitative evidence syntheses (QES), are becoming more commonly used in guideline development [20]. To our knowledge, no previous study examined the providers nor patients’ perspectives on monitoring using imaging post-recovery. Thus, the evidence we brought in this primary qualitative study provided a wealth of knowledge in great breadth and depth that would have not been possible otherwise. Further, we made every effort to maximize representation from different parts of the world. We included providers and patients from most continents, hence capturing a global perspective on the contextual factors influencing the implementation of monitoring using imaging. We conducted this qualitative research using at most considerations for trustworthiness including credibility, reflexivity, and confirmability. More importantly, the findings of this study were presented alongside evidence on the interventions’ benefits and harms to the guideline development guide and eventually incorporated into the COVID-19 living catalogue of guidelines [9].

Challenges encountered included our ability to recruit more patients. Most providers indicated that they needed ethical approval from their own health institutions to refer patients to us. Those patients who were referred by providers were mainly health care providers themselves. However, we relied on a patient support group to refer patients to be interviewed in order to maximize participation. In a few cases, language used during the interview was a challenge. Not all individuals were fluent in English. In one case, the patient had to call in the sister to translate the views. The vast majority were able to articulate freely.

Another challenge was recruiting providers from all specialties. For example, our sample did not include oncologists nor cardiologists. Perhaps, their perspectives would have concurred with our findings but also expanded.

## Conclusion

This study explored the different contextual factors influencing the use of chest imaging in COVID-19 patients for post-recovery monitoring using the evidence to practice framework. This study demonstrated that there is a high value and acceptability using imaging but there were factors influencing feasibility, equity and some practical considerations associated with implementation. We suggested several measures to improve the feasibility of the guideline such as using validated risk score predictive tools for lung complications to recommend the appropriate imaging modality and complementary pulmonary function test.

### Supplementary Information

**Additional file 1: Appendix 1.** KI interview guide for providers.

**Additional file 2: Appendix 2.** KI interview guide for patients.

**Additional file 3: Appendix 3.** Participants’ list: providers and patients.

**Additional file 4: Appendix 4.** COREQ checklist.

**Additional file 5: Appendix 5.** Valuation of outcomes associated with using chest imaging to monitor COVID-19 pulmonary sequelae, with exemplary quotes.** Appendix 6.** Preferences for each chest imaging modality used to monitor COVID-19 pulmonary sequelae, by indication, pros and cons, with exemplary quotes.** Appendix 7.** Acceptability of using chest imaging to monitor COVID-19 pulmonary sequelae, by providers and patients respectively, its determinants, with exemplary quotes.** Appendix 8.** Determinants of equity of using chest imaging to monitor COVID-19 pulmonary sequelae and exemplary quotes.** Appendix 9.** Feasibility of using chest imaging to monitor COVID-19 pulmonary sequelae by facilitators and barriers, with exemplary quotes.** Appendix 10.** Practical issues that patients might consider when using chest imaging to monitor COVID-19 pulmonary sequelae, with exemplary quotes.

## Data Availability

The datasets analyzed during the current study are available from the corresponding author on request.

## Abbreviations

COVID-19: Coronavirus disease
WHO: World Health Organization
ICU: Intensive care unit
CT scan: Computed tomography scan
EtD: GRADE evidence to decision
DLCO: Diffusing capacity of lung for carbon monoxide
