# Supplementary material for: Understanding contextual and practical factors to inform WHO recommendations on using chest imaging to monitor COVID-19 pulmonary sequelae: a qualitative study exploring stakeholders’ perspective
Source: Health Res Policy Syst. 2024 Jun 11;22:67. doi: 10.1186/s12961-023-01088-1 (PMC11167887; doi:10.1186/s12961-023-01088-1)
Supplement: Supplementary file 1 — Additional file 1: Appendix 1. KI interview guide for providers. [file 12961_2023_1088_MOESM1_ESM.docx]

Appendix 1- Interview guide- Providers

| **CONSTRUCT** | **QUESTIONS** |
| --- | --- |
| **VALUES** | How important it is to use imaging for COVID-19 survivors? And for what reasons? |
| **PREFERENCES** | Which test is more likely to provide better results and in which health conditions? X-ray, CT scan, ultrasound? |
| **CURRENT PRACTICE** | Describe the process of monitoring COVID-19 survivors in your practice (frequency, tests etc. ) |
| **ACCEPTABILITY** | How do patients accept this type of monitoring? Their tolerance for the tests? |
| **FEASIBILITY** | Are there enough resources in your organization to do those tests including: cost of supplies, human resources, and enough staff to remind patients, report results, follow-up for the results  How disruptive the monitoring is for other clinical activities? |
| **EQUITY** | Are there groups of individuals that may be at a disadvantage for receiving any of those tests? For what reasons (cost, underlying clinical conditions, gender, accessibility)  What can be done to overcome those challenges? |
| **PRACTICAL CONSIDERATIONS** | Facilitators/ barriers: time, transportation, number of visits, pregnancy, nursing, emotional |
