# Supplementary material for: Understanding contextual and practical factors to inform WHO recommendations on using chest imaging to monitor COVID-19 pulmonary sequelae: a qualitative study exploring stakeholders’ perspective
Source: Health Res Policy Syst. 2024 Jun 11;22:67. doi: 10.1186/s12961-023-01088-1 (PMC11167887; doi:10.1186/s12961-023-01088-1)
Supplement: Supplementary file 2 — Additional file 2: Appendix 2. KI interview guide for patients. [file 12961_2023_1088_MOESM2_ESM.docx]

Appendix 2- Interview guide- Patients

| **CONSTRUCT** | **QUESTIONS** |
| --- | --- |
| **EXPERIENCE** | Tell me about your health condition after discharge from hospital (what type of symptoms you were having, how difficult your everyday life was after COVID-19)  Were you asked to do a chest imaging test after you recovered? What test did you receive? |
| **ACCEPTABILITY** | Describe your physical condition when you went to the clinic to do the test?  Tell me about your experience at the clinic: health care provider provided you enough instructions about the test and why you are doing it? What about the results, were they conveyed to you in a timely manner? Clear instructions? |
| **FEASIBILITY** | How disruptive was going to do the test to your daily life?  What challenges you faced undergoing the test? |
| **EQUITY** | What about challenges accessing the test? (cost, understanding the recommendation, did you need to consent to do the test, unfamiliarity with the setting, language ) |
| **PRACTICAL**  **CONSIDERATIONS** | Facilitators/ Barriers: Were you concerned about the number of visits to the clinic? taking time off from work to visit the clinic? the commute and transportation, any issues around those? What about your emotional status? Any pregnancy or nursing issues to worry about for radiation exposure? |
