# Supplementary material for: Understanding contextual and practical factors to inform WHO recommendations on using chest imaging to monitor COVID-19 pulmonary sequelae: a qualitative study exploring stakeholders’ perspective
Source: Health Res Policy Syst. 2024 Jun 11;22:67. doi: 10.1186/s12961-023-01088-1 (PMC11167887; doi:10.1186/s12961-023-01088-1)
Supplement: Supplementary file 3 — Additional file 3: Appendix 3. Participants’ list: providers and patients. [file 12961_2023_1088_MOESM3_ESM.doc]

Appendix 3: Participant list

1. Providers

| No | Code name | Country | Specialty | Date |
| --- | --- | --- | --- | --- |
| 1 | Swiss-P02 | Switzerland | Nephrologist | November 12 |
| 2 | US-P03 | USA | Pulmonologist | November 3 |
| 3 | Jordan-P04 | Jordan | Pulmonologist | November 11 |
| 4 | India-P05 | India | Radiologist | November 13 |
| 5 | Nigeria-P06 | Nigeria | Radiologist | November 13 |
| 6 | Ethiopia-P07 | Ethiopia | Pulmonologist | November 14 |
| 7 | US-P08 | USA | Pulmonologist | November 14 |
| 8 | India-P09 | India | Nephrologist | November 15 |
| 9 | Ethiopia-P10 | Ethiopia | Pulmonologist | November 15 |
| 10 | Cameroon-P12 | Cameroon | General practitioner | November 16 |
| 11 | India-P13 | India | Pulmonologist | November 18 |
| 12 | South Africa-P14 | South Africa | Pulmonologist | November 20 |
| 13 | India-P15 | India | Nephrologist | November 18 |
| 14 | South Africa-P16 | South Africa | Radiologist | November 19 |
| 15 | South Africa-P17 | South Africa | Intensivist | November 19 |
| 16 | Serbia-P18 | Serbia | Pulmonologist | November 20 |
| 17 | Serbia-P19 | Serbia | Pulmonologist | November 20 |
| 18 | India-P20 | India | Pulmonologist | November 20 |
| 19 | Italy-P21 | Italy | Radiologist | November 17 |
| 20 | Italy-P22 | Italy | Family Medicine | November 21 |
| 21 | Italy-P23 | Italy | Radiologist | November 16 |
| 22 (patient) | Ethiopia-P11)/ Pa03 | Ethiopia | Patient /Pulmonology fellow | November 15 |
| 23 | Lebanon-P24 | Lebanon | Intensivist | November 29 |
| 24 | Spain-P25 | Spain | Pulmonologist | December 16 |

Patients

| No | Codename | Country | Date |
| --- | --- | --- | --- |
| 1 | US-Pa01 | USA | November 4 |
| 2 | Africa-Pa02 | Cote D’ivoire | November 5 |
| 3 | Pakistan-Pa04 | Pakistan | November 20 |
| 4 | Czech-Pa05 | Czech republic | November 20 |
| 5 | Czech-Pa06 | Czech Republic | November 20 |
| 6 | Pakistan-Pa07 | Pakistan | November 21 |
| 7 | India-Pa08 | India | November 21 |
| 8/provider | Ethiopia-P11)/ Pa03 | Ethiopia | November 15 |
| 9 | Italy –Pa09 | Italy | November 18 |
| 10 | Spain-Pa11 | Spain | December 26 |
