# Supplementary material for: Understanding contextual and practical factors to inform WHO recommendations on using chest imaging to monitor COVID-19 pulmonary sequelae: a qualitative study exploring stakeholders’ perspective
Source: Health Res Policy Syst. 2024 Jun 11;22:67. doi: 10.1186/s12961-023-01088-1 (PMC11167887; doi:10.1186/s12961-023-01088-1)
Supplement: Supplementary file 5 — Additional file 5: Appendix 5. Valuation of outcomes associated with using chest imaging to monitor COVID-19 pulmonary sequelae, with exemplary quotes. Appendix 6. Preferences for each chest imaging modality used to monitor COVID-19 pulmonary sequelae, by indication, pros and cons, with exemplary quotes. Appendix 7. Acceptability of using chest imaging to monitor COVID-19 pulmonary sequelae, by providers and patients respectively, its determinants, with exemplary quotes. Appendix 8. Determinants of equity of using chest imaging to monitor COVID-19 pulmonary sequelae and exemplary quotes. Appendix 9. Feasibility of using chest imaging to monitor COVID-19 pulmonary sequelae by facilitators and barriers, with exemplary quotes. Appendix 10. Practical issues that patients might consider when using chest imaging to monitor COVID-19 pulmonary sequelae, with exemplary quotes. [file 12961_2023_1088_MOESM5_ESM.docx]

Appendix 5- Valuation of outcomes associated with using chest imaging to monitor COVID-19 pulmonary sequelae, with exemplary quotes.

| Values | Outcomes valued | Exemplary quotes |
| --- | --- | --- |
| Highly valuable when chosen wisely for | *Perceived need for monitoring and follow-up* | ***Nigeria- P06:*** *It all depends on the case, it’s case by case but the first 4 weeks of follow up are crucial.*  ***Ethiopia- P11:*** *I think also the follow up is very important and we need to communicate with them.* |
|  | *Imaging modality decision is driven by patients’ status: during hospitalization, upon discharge, persistence of symptoms post-recovery, and clinical findings after the first visit* | ***India-P13:*** *So it depends on the severity of the disease during the hospitalization, and the condition of the patient on discharge.*  ***India-P05: ...*** *uh,* ***there are certain guidelines****, which, they have been, you know, advised that after your discharge also****, if any, you know, symptoms, visible or filled by the patients, they are having any shortness of breath after discharge or they are having any difficulty with relation to respiration, or any other system related illness, or if there were any comorbidities and such, they have to report back*** *either to the same hospital and that is to us or any hospital, which is nearby to them, within their reach, within 7 days of the start of symptoms. And based on the presentation of the patient, the follow up is decided. If he's up 60 years of age, he's a high risk patient, or she is a high risk patient, then a stringent follow up is taken.*  ***Pakistan-Pa07:*** *Doctor told me if you feel anything let us know we will do a chest radiography* |
|  | *Monitoring progression* | ***South Africa-P16:*** *We're seeing people who just take longer to recover. We think patients who come back for follow up and have persistent respiratory symptoms. I think with our setup here in particular that we have a very high burden of illness. We are almost in a TB endemic area. We have the highest incidence of tuberculosis. We also have a very high incidence of HIV so we have a lot of confounding factors with our patients. So, for example, just had to request that* ***patient who is known to have a baseline interstitial lung disease have a routine follow up. He had been tested positive for COVID and now was experiencing a deterioration in his baseline. And for that reason, he had a high resolution CT scan requested to try and troubleshoot. Is this just his baseline, interstitial lung disease that's deteriorating? Or has he in fact undergone? Is this a COVID related abnormality? So we have those sorts of problems.*** |
|  | *Early detection of new diseases* | ***Ethiopia- P07:*** *Definitely. The severity of COVID increases from severe to critical. There was a study that showed that at least half of them, you know, 20 of the 40 patients on whom they took respiratory samples for culture, they were able* ***to demonstrate coinfection pneumonia and something like that. So that's common also and the imaging will help to catch such additional problems too.*** |
|  | *Decreasing risk of mortality from opportunistic diseases* | ***Nigeria- P06: The follow up is major. Some of them have a low immunity and if we do not catch the bacterial infection on time yes we lost them. I think the follow up is very crucial.*** |
|  | *Scientific purpose: understanding the long-term sequelae of the infection* | ***US-P08****: mostly to understand the disease more. You see what they have findings on the imaging initially they have been improving.* ***Also mostly to kind of look at long term sequela of the condition. But mostly to understand the disease itself, which is kind of relatively had been new.*** |
| When clinically not indicated, it is considered unethical and overused | *When clinically not indicated (e.g., mild cases), it is highly unethical to expose patients to unnecessary radiation* | ***India-P09:*** *if the patient is clinically doing well, I mean, is improving, If a person functional capacity is better, if he was dependent on oxygen but then after discharge, we have a resolution of Covid-19 and he is clinically going well, then I don't think that doing a CT scan will add to the decision making in terms of therapy.* ***That is what my personal belief is, but yes, obviously, you don't want them to be having unnecessary exposure to radiation with multiples radiographs or multiple CT scans.*** |
|  | *May lead to unnecessary findings* | ***Swiss-P02:*** *I think so, and then you might have a problem that* ***you start to find incidental findings. And then you're going to have to deal with following up on other things that you might see on the X ray that may not be even real, you know, and that's another ethical problem. If they don't need the radiography, you're exposing them to radiation*** |

Appendix 6: Preferences for each chest imaging modality used to monitor COVID-19 pulmonary sequelae, by indication, pros and cons, with exemplary quotes

| Test modality |  | Exemplary quotes |
| --- | --- | --- |
| Chest radiography | | |
| Indication | *Mild cases still complaining of chest pain*  *Monitor clearance of lungs*  *For dialysis patients to detect water retention* | ***Swiss-P02:*** *I think honestly, for our patients, sometimes the X rays actually helpful. Because sometimes they lose a lot of weight and we need to know how much water we need to remove from the patient in a dialysis treatment. And occasionally you don't know how quickly the patient has lost weight with COVID. So, sometimes you do the X Ray with an excuse of COVID, but you're actually looking to see, are they filling up with water, are they're going to develop heart failure. So, for us, sometimes there's another extra added value* |
| Chest CT | | |
| Indication | *Identify fibrotic changes*  *Detect additional diseases*  *For patients depending on oxygen long after discharge* | ***India- P05:*** *as far as the CT scan is concerned, it is basically, it gives you a fuller picture of the chest. I mean, what is happening inside the lungs, right from the high limb, to the trachea to the lung parenchyma. So generally, the mindset for patients out here is better go for a CT scan because, uh, it gives a good resolution.* |
| Lung ultrasound | | |
| Indication | *Peripheral consolidation only* | ***US-P08:*** *... We’ve been relying a lot on it and they're criteria to follow with the ultrasound. It's a very easy modality. Yes, we're using it. And now it's like, you have those small portable ones. It's very easy to clean it from room after room, way better than cleaning the whole chest X Ray machine.* |

Appendix 7- Acceptability of using chest imaging to monitor COVID-19 pulmonary sequelae, by providers and patients respectively, its determinants, with exemplary quotes

| Acceptability | Determinants of acceptability | Exemplary quotes |
| --- | --- | --- |
| By providers | *Monitoring is a common practice well received by patients* | ***India- P09:*** *So, usually after the patients are discharged, we usually call them within 7 days for the first visit. In that visit we monitor them clinically. We usually do not repeat the radiography. After 7 days we ask them to visit after 21 days and then usually, 21 days after, we usually repeat the radiographs. And then, if there are not clinically improving as we expected, then we do a CT scan.*  ***Ethiopia-P10:*** *Yeah, because COVID is a disease still not well known by the community and even by the health care professionals, whatever I council them, they assume I am the expert and so whatever I council them, they accept. I have never seen resistance so far.* |
|  | *Patients accustomed to monitoring* | ***India-P09:*** *Yeah, the nephrology problems, obviously patient with CPD [chronic pulmonary disease] and with those on dialysis, even other renal diseases like lupus nephritis and other kidney problems, they usually follow up with us routinely also.* |
| By patients | *Trusting providers* | ***Czech-Pa06:*** *You know, I reacted more to things, and my doctor said it can be the immune system that is more reactive now, but I wanted just, like, wanted to be sure that everything is okay. But the results of spirometry were okay, so I trust my doctor. Yeah. I trust my doctor. Yeah.*  ***Africa-Pa02:*** *So I stayed for 5 days at the hospital to treat both Covid-19 and diabetes….After 22 days, I repeated the CT scan and still my lungs were a bit affected so he said let’s continue the treatment for 15 more days. I did that.* |
|  | *Affordability* | ***Interviewer:*** *Do you think all the population is able to do what you were able to do?*  ***Africa-Pa02:*** *No because it is very expensive, both CT scan and treatment… Just for you to have an idea, the CT scan alone costs 160$ every time.*  ***Ethiopia-P07:*** *But my worry is that because most of our patients are paying to of pocket, they don't have a well-established health insurance system…. They will pay, like, for example, for CT scan without contrast, if it’s with contrast, it doubles right away and if it’s without contrast, like, it is 15 to 20 $.... If they are convinced that it helps them, they will come but the problem is that most of our patients, they may not be able to pay for the CT scan.* |
|  | *Anxiety induced by testing* | ***Interviewer****: And also the anxiety. Let’s say you’re waiting for a CT scan result, waiting to see how the disease is progressing, how anxious would you be?*  ***Ethiopia-Pa03:*** *Yeah definitely I would be very anxious because you really don’t know what will happen, you really don’t know the sequela of the disease. Pathology might persist for a long time and it also might lead to a risk of other pathogens to cause other disease, my lung function might be deteriorating, I might have long term sequela.* |

Appendix 8- Determinants of equity of using chest imaging to monitor COVID-19 pulmonary sequelae and exemplary quotes

| Equity | Determinants of equity | Exemplary quotes |
| --- | --- | --- |
| Across countries | *Access to resources varied between developing and developed* | ***US-P08:*** *So, here in South Carolina, like, um, we have, uh, the biggest hospital, I think it's a medical University of South Carolina in Charleston. We have 2 big centers, which I'm part of one of them and there are any small affiliated hospitals. CT scan is available very widely. Any issues that needs any further care, again, we have video system, so if they need further evaluation, they go to their closest hospital, they get stabilized and they get transferred to one of the biggest centers, which is not too far. And either they get transferred by ground, or they're sick, they get transferred by air, which is really fast, like talking 10 to 15 minutes by helicopter or a plane. So the CT scan is widely available if it needs, like, a little bit of more advanced things the biggest centers, definitely, the patients get referred to them, but mostly when they get sick and need admission. For outpatient, like, imaging, scans, pulmonary function test, ultrasounds, these things are very widely available.*  Compared with what providers in Africa said:  ***Cameroon-P11:*** *Okay so I would think it’s very important to do radiological follow up…Most of our district health facilities, will not have radiology equipment. So normally we would just do auscultations, and manage them as such, and when they go we do not follow up.* |
|  | *Countries with and without government health coverage* | ***Jordan- P04:*** *No, it's actually all cancer patients [also affected with COVID] in Jordan are covered by the government so the government will end up paying for their treatment and follow up and all what they need until they are done. And even beyond that even.*  ***Interviewer:*** *So, they won't be an equity issue in terms of accessing the services.*  ***Jordan-P04:*** *Not at all*  ***Czech-Pa06:*** *it's covered by the health insurance. Okay. So everyone is covered, everybody in the Czech Republic*  ***US-P03:*** *Many of our hospital, like, the majority of our hospitalized COVID patients are over the age of 65 so they're mostly Medicare, so most of them have government insurance and then a fair amount of Medicaid. Here specifically at our hospital, we have a program that if you have no insurance, and it's a hospital-based service, like radiology, the hospital will subsidize it. You have to jump through some hoops to show that you have that need*  This is compared to a participant from Africa who said:  ***Africa-Pa01:*** *Because it is very expensive, both CT scan and treatment. They [uninsured] would die. Just for you to have an idea, the CT scan alone costs 160$ every time.* |
| Within countries | *Insurance coverage varied by individuals* | ***Pakistan-Pa04:*** *I am a teacher so my insurance company is taking care of my entire cost of stay in hospital and all expenses…Not the case for everyone.. We are a third world country. For example, Remdesivir many cannot afford it. CT scan is slightly expensive. 75% will not be able to afford those expenses. In wave 1, people could not afford the hospitalization so ended being very sick.* |
|  | *Quality of services lower for the public sector* | ***Nigeria-P06:*** *Um, yes although the state government they have a center that is funded by the government, so those who can’t afford are referred to them. But when they come here to our hospital and they can’t afford, they go there.*  ***India- P09:*** *So, CT scan facilities are most of the time available in, at least 2 or 3 cities like ours. Public sector it is a few. It is difficult to access. In private sectors, there are lots of CT scans, but again, the out of expenditure really goes high.*  ***Pakistan-Pa04:*** *I went to a private hospital, it is a better service.. I had a smooth recovery because of the private hospital service. The doctors take care of you and keep in touch with you. This makes it easier. Mostly who cannot afford it go to public hospital. Public hospitals are there is a big gap between these two that need to be filled up… During the peak, the public hospitals used parking lots etc.* |
|  | *Urban vs rural* | ***Ethiopia- P07:*** *In some cities? In some cities only, in the bigger cities only. We have like 10 regions in the country, with Addis Ababa as the capital city and it is full of CT scans. You can find it in almost all hospitals, particularly in the private ones. As for the rest, probably 5 to 6 of the regions have it but the rest they don’t have it.*    ***India- P05:*** *Yeah, that can be a problem because the means of travel are, you know, buses and trains in our country for traveling from one area to another and areas who have radiological imaging modalities are mostly located in urban areas and suburban areas. So, ours basically is a rural hospital, which is situated nearby a city. So, we are like a tertiary care hospital for such patients where multiple people from villages and multiple rural areas, uh, you know, confluence for, uh, such CT scans. So, we monitor we cater to the patients, which are, uh, from, uh, areas around the city around our area.*  ***Nigeria-P06:*** *Yeah, yes accessibility would be another issue because if someone is living in the village nearby they can come to the hospital immediately. So yes accessibility is an important factor. People who are living far in villages it would be more difficult for them to come to the hospital. And we don’t have hospitals all over the place.* |
| *Non-COVID-19 patients* |  | ***South- Africa-P16:*** *Not in our institution specifically in the pandemic when we were probably doing much more radiographs on inpatients. We had emptied a lot of our wards and turn them into COVID wards. A lot of radiographs with mobile x-rays units have been done, but that was counterbalance by the fact that our hospital was closed for a lot of routine work. So, a lot of oncology, maybe not quality, went on as usual, but a lot of the routine clinical clinics, like the orthopedics clinics and stuff. A lot of those fell away*  ***India-P09:*** *Yeah, so definitely COVID patients are still able to access healthcare in an appropriate period. And it is most unfortunate, those patients who have non-COVID illness, they are much more neglected, uh, because of this whole crisis. First of all, it's like, patients see that the hospital where COVID patients are treated, it's a COVID hospital and, even if they have a problem, which is like, for example traffic accident, they would go to a non-COVID hospital, which is not much. Most of the good hospitals who have good facilities are now COVID hospitals. They are not coming to us because they are being scared that this is a COVID hospital and that they may end up, uh, getting it from the hospital, although probably they would have got much more benefit if they have come to our hospital earlier.* |

Appendix 9- Feasibility of using chest imaging to monitor COVID-19 pulmonary sequelae by facilitators and barriers, with exemplary quotes

| Feasibility |  | Exemplary quotes |
| --- | --- | --- |
| Facilitators | *Having a post-recovery COVID-19 unit and protocols* | ***Ethiopia-P07:*** *We have a clinic, which is, uh, you know, we have a head nurse. We have a few. 6 to 7 nurses and because we have limited number of nurses, this group of nurses, half of the time they spend it with the chest team.. When they come to the clinic, and we see like, 45 to 50 patients and that divided to like on average 5 residents, 1 fellow and 1 senior. We have to go through each CT scan of each patient.* |
|  | *Annexing it to an existing monitoring system for other diseases* | ***Jordan- P04:*** *It will actually be going to be easily integrated because a lot of these patients [oncology patients] they end up getting CT scans and, you know, pet scans routinely. Yeah, because as part of their, you know, follow up and as part of their staging, so I don't think it will be a problem at all. .. What we can do, and we've been doing that is to try and combine these tests together so that if they have 1 appointment, they can get the rest of it together.*  **Swiss-P02:** *They [dialysis patients] always come 3 times a week. Yeah so, for them, it's slightly different. They're used to, you know, we can do a lot during the dialysis* |
|  | *Ability to scale up the efforts* | ***India-P05:*** *Daily, we are able to see around 75 to 100 patients…what we have done in our Institute, that we have a hand, picked a few consultants from my department myself included. So, every other day, and, I mean, every alternate day, we are having emergency duties. So we are having dedicated duties for reporting COVID patients, on CT scan and we do it, uh, every alternate day…I mean, it has increased the burden for sure. But then we are doing it.* |
| Barriers | *Being too deconditioned to go for a test following a severe illness* | ***US-Pa01****: I had to be lying in bed for 3 weeks. So after that, I literally could not stand up. I wasn't able to sort of get out of the bed into a wheelchair to go to the bathroom for a week. We're talking about a severe case. You know, it's also how long have you been in the hospital?… Mostly getting out of the house to get to get to an appointment. The energy that you need to get out, you know, having the help that you need to kind of have an invitation to get there.* |
|  | *Not enough specialists in developing countries* | ***South Africa-P14:*** *the lack of specialists to follow these patients. I mean, South Africa is a country of 65M people and we've got 70 pulmonologists.*  ***Ethiopia-P 11 (Pa03):*** *We have very few pulmonologists, like 7 or 9. Private we are few. Like total not more than 15. Imagine in this big country, with like 100 million people, it’s hard.* |
|  | *Not enough dedicated staff responsible for tracing patients* | ***US-P03:*** *I think the biggest challenge, obviously there's a huge number of patients. It is overwhelming like, we've created a small group of doctors to, like, call patients and tell them they're COVID positive and discuss the results. But eventually we were overwhelmed because there just so many positive patients. And so the solution was to then just say, well, you should talk to your ordering provider, your primary provider. The hard part there is as much as everyone is trying to keep up with, things changed so quickly with COVID recommendations, that primary care providers who are giving the right advice 6 weeks ago are no longer giving you the right advice.* |
|  | *Back log in imaging appointment due to limited equipment (e.g., CT scan)* | ***Interviewer:*** *And do you have a back log of patients like people have to wait some time before they get their test done or is it something that is being done on the spot?*  ***Ethiopia-P11:*** *Yeah we have a back log. We have a lot of back log [radiology tests]. Even for admitted patients, it is difficult sometimes.*  ***South Africa-P16:*** *We have a lot of referrals [for radiology from primary health care] to our hospital and with our hospital that comes a prolonged, waiting, period often. So I'm not sure if that this is an exaggerated delay based on the fact that these patients are probably struggling to access, sort of the healthcare, or if it's true. A delay of 6 to 8 weeks. But we are seeing that sort of delay a very long time. 6 weeks, even.* |

Appendix 10- Practical issues that patients might consider when using chest imaging to monitor COVID-19 pulmonary sequelae, with exemplary quotes

| **Practical issues**  **related to the use of chest imaging to monitor those sequelae** | - *Elderly living in nursing homes or dependent on others* - *Women, nursing women, and pregnant women* - *Dialysis patients need to come early or leave late before or after dialysis for testing* - *Transportation and distance to centers particularly for patients who are deconditioned or who just recovered* - *Coordination of care: primary health care provider not communicating with other providers* - *Escort particularly for sick or older patients* |
| --- | --- |

| Practical issues |  | Exemplary quotes |
| --- | --- | --- |
| Tests and visits | *Elderly population living in nursing homes* | ***US-P03:*** *The patient population, that's a little harder to get imaging on actually patients who go to, like, nursing homes. Because many skilled nursing facilities, they don't have radiology services there and the ability to get the patient with medical transport from the skilled nursing facility to wherever the radiology is in back or the doctor's appointment, or whatever, that too is also actually very difficult. So I think that population, that group where they have both mobility and transportation issues is a big one*. |
|  | *Dialysis patients* | ***Swiss-P02:*** *I think for the dialysis patients again, for them, it's a burden because either they must come earlier to go to the radiography before the dialysis, or then go after dialysis and then it delays them going home. And then it complicates the transport.* |
| Coordination of care | *Poor coordination between providers* | ***Ethiopia-Pa03:*** *Yeah, I think so. From my side, I was communicating with the social worker from the hospital because I was a staff there so they call me and they put me in contact with the psych department….Things are very decentralized so a lot of guys might call them. The follow up is not organized so this might cause people to avoid seeking health care.*  ***Swiss- P02****: So then we have to ask the family doctor to let us know what's going on.* |
| Travel and driving | *Those dependent on other family members* | ***Nigeria-06:*** *Yes, of course, the older population will be dependent on people to assist them. It easier for the younger to come to the hospital, they have a car to go around. Some families are not nearby too. Also people with comorbidities, this will also affect how fast they can come to the hospital. It would be a major problem. Also people who are not part of the government. Some people also call and they are told to stay in their house and then paramedics come and check them out. If they fall into the criteria of a chest x ray, they would do it. Those who have access for a phone to call are fine. So yes this leads to inequalities. Those who have access come on time and get access to healthcare faster.* |
|  | *Transportation from far areas* | ***Swiss-P02****: Definitely. and for some patients, it's expensive, you know, they have to catch a train which might also be risky or a taxi and a taxi is expensive. Especially elderly people. Some of them don't drive. Their children may not be around, or they don't have anyone to drive them. This can also cost a lot of money.* |
|  | *Needing escort to and from the clinic* | ***Africa- Pa01:***  *After few treatments, I was able to drive but of course I always had someone with me. Never went alone, afraid something would happen to me. But first days and first treatments, I was not able to drive. Always had someone accompanying me.* |
| Pregnancy and nursing | *Being women* | ***South Africa- P16:*** *So, high radiation risk or high radiation dose to the breasts. And reasonably achievable. So, we try to take it down to the minimum.*  ***South Africa- P16:*** *We shield the baby that is mommy's tummy and we'll take a chest radiography.*  ***South Africa- P16:*** *Unfortunately, sometimes the condition of the mom does demand a CT scan and then we just have to make the best decision.*  ***Swiss-P02****: I think the one problem is young women with pregnancy. That's a problem. That's a huge problem.* |
